# Bridging implementation gaps to connect large ecological datasets and complex models

**DOI:** 10.1002/ece3.8420

**Published:** 2021-12-14

**Authors:** Ann M. Raiho, E. Fleur Nicklen, Adrianna C. Foster, Carl A. Roland, Mevin B. Hooten

**Affiliations:** ^1^ Department of Fish, Wildlife, and Conservation Biology Colorado State University Fort Collins Colorado USA; ^2^ Denali National Park and Preserve National Park Service Fairbanks Alaska USA; ^3^ School of Informatics, Computing, and Cyber Systems Northern Arizona University Flagstaff Arizona USA; ^4^ Department of Statistics Colorado State University Fort Collins Colorado USA; ^5^ Colorado Cooperative Fish and Wildlife Research Unit U.S. Geological Survey Fort Collins Colorado USA

**Keywords:** boreal forest, Denali National Park, dendroecology, forest gap models, model calibration, *Picea glauca*, *Picea mariana*, surrogate modeling

## Abstract

Merging robust statistical methods with complex simulation models is a frontier for improving ecological inference and forecasting. However, bringing these tools together is not always straightforward. Matching data with model output, determining starting conditions, and addressing high dimensionality are some of the complexities that arise when attempting to incorporate ecological field data with mechanistic models directly using sophisticated statistical methods. To illustrate these complexities and pragmatic paths forward, we present an analysis using tree‐ring basal area reconstructions in Denali National Park (DNPP) to constrain successional trajectories of two spruce species (*Picea mariana* and *Picea glauca*) simulated by a forest gap model, University of Virginia Forest Model Enhanced—UVAFME. Through this process, we provide preliminary ecological inference about the long‐term competitive dynamics between slow‐growing *P. mariana* and relatively faster‐growing *P. glauca*. Incorporating tree‐ring data into UVAFME allowed us to estimate a bias correction for stand age with improved parameter estimates. We found that higher parameter values for *P. mariana* minimum growth under stress and *P. glauca* maximum growth rate were key to improving simulations of coexistence, agreeing with recent research that faster‐growing *P. glauca* may outcompete *P. mariana* under climate change scenarios. The implementation challenges we highlight are a crucial part of the conversation for how to bring models together with data to improve ecological inference and forecasting.

## Graphical abstract

Combing big data with complex models is a promising path forward for solving global ecological problems. We present solutions to three underreported challenges when combing big data with models in ecology using tree‐ring data and a forest gap model in Alaska.

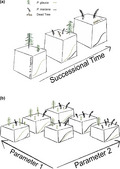

## 1 INTRODUCTION

Increasingly, computational simulations of complex individual‐level patterns are used to support ecological understanding and forecasting (Dietze et al., 2018). Simultaneously, modern statistical techniques, such as machine learning, are rapidly being developed to provide predictions using “big” data in real time (Christin et al., 2019; Luo et al., 2011; Peters et al., 2014). Computational models alone can be incomplete ecosystem representations because of possible misspecifications or omissions of processes, and machine‐learning approaches alone may not provide insights about mechanisms. Similarly, ecological field data can never fully observe an ecological system. Combining these techniques to understand mechanisms and improve predictions is a promising path forward (Reichstein et al., 2019; Wikle & Hooten, 2010). Examples of such ecological applications (Fer et al., 2018; Oberpriller et al., 2021; Pietzsch et al., 2020; Speich et al., 2021; Tao et al., 2020) are critical for moving beyond implementation barriers and solving urgent ecological problems by bringing these promising tools to larger application‐based audiences.

We focus on an urgent ecological problem in interior Alaska where changes in the environment, such as permafrost degradation (Hinzman et al., 2006; Osterkamp, 2005; Osterkamp et al., 2009; Osterkamp & Romanovsky, 1999; Sniderhan & Baltzer, 2016), have occurred over large areas and are beginning to alter boreal forest distributions (Jorgenson et al., 2001) including within national parks. In Denali National Park, Nicklen et al. (2021) found that the climate growth responses of two dominant spruce (*Picea*) species, but especially *Picea glauca*, depended on the presence of near‐surface permafrost. Warm summer conditions increased *P*. *glauca* radial growth on well‐drained soils, but strongly reduced growth when growing on near‐surface permafrost. *Picea mariana*, currently occupying permafrost terrain, may suffer range contraction under climate change as competitor *P*. *glauca* expands into more suitable terrain as permafrost thaws (ACIA, 2005; Wirth et al., 2008). Contrary to these predictions, increased low‐severity fire frequency (e.g., fraction surface carbon consumed) may allow for continued self‐replacement successional trajectories (Johnstone & Chapin, 2006; Johnstone et al., 2010; Kane et al., 2007). A comprehensive assessment of the current status of forests in Denali National Park in Roland et al. (2013) showed that *P*. *mariana* and *P*. *glauca* do not typically co‐occur. However, Roland et al. (2013) also found that sites where *P*. *glauca* were most common were also the sites where *P*. *mariana* achieved greatest diameters. This suggests that the current exclusion of *P*. *mariana* from areas dominated by *P*. *glauca* may be related to competition between the two species not necessarily niche limitations. *Picea mariana* is slow to grow early in succession (Gutsell & Johnson, 2002; Wagner & Robinson, 2006) and may be hidden from current assessments that only consider modern data for future predictions (Foster et al., 2019). However, these predictions focus on observations from the modern landscape and do not incorporate long‐term (i.e., centennial) data, which may be relevant for predicting long‐term system behavior. To disentangle this ecological problem and provide national park managers and visitors with predictions of forest response to climate change, we can improve mechanisms portrayed within ecosystem models by combining model simulations with long‐term field data.

Combining observations with computational models statistically can be formally conducted through model calibration. In purely statistical models, this process is simply referred to as “model fitting” where uncertainty in a selected model parameterization can be accounted for in a statistical context where data, process, and parameter models may be developed to make posterior parameter inference (Berliner, 1996). But, it is often computationally infeasible to embed a fully computational model within this type of statistical model directly. For computational models, many different model calibration approaches can be taken ranging from informal to formal statistical techniques. A more informal approach involves “tuning” parameters where the user alters parameters until the output more closely matches the observations. More formal techniques for model calibration involve either informing parameters directly with data (LeBauer et al., 2013) or iteratively updating parameters or parameter distributions as data become available (Fer et al., 2018; Oberpriller et al., 2021; Pietzsch et al., 2020; Speich et al., 2021; Tao et al., 2020). Similarly, modelers may choose to calibrate all parameters in a computational model, which we refer to as “full model calibration,” or a modeler can choose to focus on the most sensitive model parameters using an “a priori” parameter sensitivity analysis.

In this study, we perform calibration of an agent‐based model focusing on the most sensitive parameters. Agent‐based models are computational models used in disease, animal, forest ecology, and beyond to simulate the patterns of individuals, then calculate how the aggregation of those individual patterns leads to emergent system properties (Banks & Hooten, 2021; Hooten et al., 2020). For example, human mobility in an agent‐based model may lead to population‐level spread of a disease. Connecting data with individual‐based models can be difficult because it is unclear how the individual data relate to the emergent process of interest, such as vector‐borne disease spread among a population (Perkins et al., 2019). In agent‐based models of human mobility, data are not typically used directly to constrain the human movement process itself, but instead data can be used to constrain parameters that drive the spread of the disease between individual agents (Venkatramanan et al., 2018). Similarly, in forest ecology, data collected at the individual tree level can be used to constrain growth parameters in individual‐based models, which leads to an improved understanding of long‐term forest change.

Constraining mechanistic ecological simulation model parameters, however, remains challenging. Mechanistic ecological simulators often have detailed process representations that are aimed at improving realism at the cost of making models computationally expensive. Parallelization of model calculations can greatly reduce computational times. Yet, during model development stages, especially for the non‐specialist, this is often not a worthwhile solution because it requires a large investment in computing resources and development of parallelization or simulation code. In this instance, we consider a “non‐specialist” a person who could expand model‐based ecological interpretation and hypothesis testing but does not have any experience with simulation modeling (Fer et al., 2021). A “non‐specialist” may be an expert in another area that is needed to improve model simulation capabilities, but a “non‐specialist” does not specialize in the simulation model code or implementation itself (e.g., a formally trained statistician or forest ecologist). Software packages exist that remove both computational and statistical barriers for performing ecological model calibration as a non‐specialist (Fer et al., 2018). However, this software, while integral to model calibration research, is in early stages of development, has yet to be applied in a broad ecological context, and is therefore not applicable to all branches of ecology. Software packages exist that remove statistical barriers (Hartig et al., 2019; Speich et al., 2021), but these packages require the user to perform the model simulations and make consequential decisions about the experimental design of the model simulations. To solve complex ecological problems, ecologists should be involved in the simulation modeling improvement process and more examples from branches of ecology using a variety of statistical methods are needed.

Approximating the computer model using a statistical surrogate or emulator has become a popular solution to model calibration (Gramacy, 2020). Surrogates are statistical representations of computer models and are developed using the paired input and output from a computer model to train a statistical surrogate that is used in the calibration process. There are many types of statistical models that can be used as surrogates such as deep neural networks, generalized additive models, and Gaussian process models. Gaussian process models (GPs) have become popular because GPs are highly accurate at out‐of‐sample prediction, allow for uncertainty quantification, and are analytically tractable (Bijak et al., 2013; Heitmann et al., 2013; Santner et al., 2018; Verrelst et al., 2016). GPs have been used as successful computer model surrogates in many contexts including engineering, physics, and Earth system modeling. Only recently have they been proposed as a useful framework for calibration of agent‐based models in ecology (Banks & Hooten, 2021; Hooten et al., 2020). Furthermore, it has been shown that GPs can account for model structural error and model parameter error simultaneously (Oberpriller et al., 2021), offering an advantage to conventional model calibration techniques (Hartig et al., 2011). As GPs become increasingly popular, many statistical software packages built for the non‐specialist are being developed (Erickson et al., 2018; Fer et al., 2018; Liu et al., 2020). While these packages remove implementation barriers, some statistical choices may not be obvious for a non‐specialist because they are based on either open scientific questions or practical rules of the model calibration process.

The non‐specialist may have to make several choices about how to create the simulations or morph existing simulation output to match the data‐generating process. These choices affect the parameter estimates and are rarely discussed. To highlight these challenges in an accessible context, we present an example using tree‐ring data collected in Denali National Park to calibrate species parameters in a state‐of‐the‐art individual‐based forest model (University of Virginia Forest Model Enhanced, UVAFME; Foster et al., 2019) illustrating a method not commonly used in ecology that may connect an ecological computer model with field data using a Gaussian process surrogate. This example demonstrates the complexities of three statistical modeling choices that are common in many ecological settings: (1) matching the field observations with model output, (2) determining the correct model start time (i.e., initial conditions) from data, and (3) fitting a high‐dimensional Gaussian process surrogate to calibrate the model parameters. This example based on UVAFME involves forest succession, a foundational ecological process, in a region where competitive relationships between species may drive biome‐level changes (Wirth et al., 2008). Successional trajectories can be observed in tree‐ring data but are seldom used in conjunction with models of succession because of the issues listed above. We calibrate UVAFME using tree‐ring‐derived basal area to gain meaningful ecological inference and show how we pragmatically overcome the three aforementioned issues in the procedure.

## 2 MATERIALS AND METHODS

### 2.1 Study area and tree‐ring data

Denali National Park (DNPP) comprises a variety of habitats because of wide elevation and topographic gradients (i.e., slopes and aspects). Tree species in DNPP have unique life history characteristics and thus occupy largely distinct habitats with a few exceptions. The most common tree species in DNPP is *Picea glauca*, followed by *Picea mariana*. *Picea glauca* is a relatively faster‐growing species that typically occupies well‐drained sites with warmer, more basic, and deeply thawed soils, while *P*. *mariana* tends to occupy areas influenced by near‐surface permafrost with thin active layers and cold, wet, acidic soils (Islam et al., 2003; Roland et al., 2013; Van Cleve et al., 1983). Forests in DNPP are under increasing pressure from climate change as temperatures, growing season length, and disturbance frequency and severity increase (Kelly et al., 2013; Roland et al., 2019). These accelerated changes have resulted in predictions that the boreal forest, including DNPP, is at the edge of a biome‐level tipping point (Scheffer et al., 2012) where water‐limited spruce‐dominated forests could be replaced by post‐fire early recruiting broadleaf‐dominated forests. Landscape features, such as organic matter depth or wetness rating, are seldom directly considered in regional predictions of species distributions and may offer refugia or buffer from extreme changes (Raiho et al., In Revision; Scharf et al., 2021).

To predict future distributions of *P*. *mariana* and *P*. *glauca*, we need a mechanistic understanding of the competitive relationship between the two species informed by long‐term data. Because Denali National Park occupies a large region (~4.7 million acres), data for model calibration are typically collected from satellite observations or from field sites along roads. However, these modern snapshot data do not hold information about successional trajectories or competitive dynamics between species. Tree‐ring data are well suited to help us gain insights about forest succession (Fritts & Swetnam, 1989). The National Park Service collected tree rings for *P*. *glauca* and *P*. *mariana* across a wide spatial domain in Denali at 233 sites. These records average 103.2 years long (SD = 49.7) and 2.6 cored trees (SD = 1.5) per site. Plot and tree sampling methods are described in Nicklen et al. (2016). We measured growth rings to 0.001 mm using either CooRecorder (Cybis Elektronik & Data AB), WinDENDRO (Regent Instruments Inc.), or a sliding scale. Ring widths were visually cross‐dated using CDendro 8.1 (Cybis Elektronik & Data AB), and cross‐dating was validated with COFECHA 12K XP (Holmes, 1983). Basal area increment, the estimated area of wood produced by each tree in each year of growth, was calculated using ring widths and tree radius with the outside‐in approach (Biondi, 1999; Johnson & Abrams, 2009). We used the sum of the ring widths as the radius unless the measured tree radius ((diameter at core/2) − average bark width) was greater than ring width sum, in which case we used the measured radius. Cores with missing outer rings were not used. We use species‐level total basal area, which was calculated by summing across the basal area increment measurements by year and species at each plot. For comparison between the tree‐ring and model simulation datasets, we remove small trees from UVAFME simulations and perform the same basal area calculation by summing across basal area increment within UVAFME outputs. Across 233 sites, there are only 10 sites that contain tree core data from at least one individual of each species (Figure A9: Appendix S1). We refer to these sites as the “coexistence sites” and focus our model calibration on those sites for a multi‐species simulation model calibration.

Tree rings are critical for observing long‐term forest succession at a particular location. However, there are many uncertainties associated with tree‐ring basal area reconstructions (Alexander et al., 2018). In particular, tree‐ring accuracy is reduced for earlier dates because only the trees that are alive at present were cored and do not represent trees that may have died in the past in the same location. This is known as the “fading record” because there is less information in the earlier tree rings about the forest stand (Babst et al., 2014; Nehrbass‐Ahles et al., 2014). While this problem is specific to tree‐ring data, we highlight it in this manuscript to show how this type of model‐data mismatch can be overcome.

### 2.2 Individual‐based forest simulation model

University of Virginia Forest Model Enhanced (UVAFME) is a forest gap model that was originally developed as an enhancement for alpine and boreal forests on previous forest gap models (Shugart et al., 2020). The basic principle underlying UVAFME is competition for light, water, and nutrients between individual trees. Changing demographics of individual trees then feeds back to the ecosystem states to alter the availability of light, water, and nutrients in the following years. Each year, individual trees are limited by the least of these factors, resulting in realistic forest stand dynamics over successional timescales as limiting factors change over time. UVAFME has been applied in the boreal forest (Yan & Shugart, 2005) and has been further extended to simulate boreal forest dynamics across regions (Foster et al., 2019). Most notably, UVAFME has the ability to simulate permafrost change over time, which greatly affects forest stand development from individual tree to regional spatial levels in Alaska (Shur & Jorgenson, 2007). Permafrost is parameterized in UVAFME as a limiting growth factor that affects tree species differently if they are parameterized as tolerant or intolerant. The current parameterization of permafrost includes fixed rates for tolerant or intolerant growth response to active layer depth. Because growth response to active layer depth is not well known at the individual tree level, we incorporated these rates as unknown parameters in our sensitivity analysis described in the upcoming section. There are 23 species‐specific parameters in UVAFME. The default parameterization for the boreal region of Alaska is fully described in Foster et al. (2019). We used site condition settings from the plot‐level data collected in Roland et al. (2019). Forest gap models have been developed to represent the expected long‐term (i.e., centennial to millennial) forest community dynamics given climate normal conditions (Bugmann, 2001). Following common forest gap model implementation, we used the default climate subroutines available within UVAFME and drew random monthly temperature and precipitation from climate normal distributions (Group, 2009, 2020). Site‐level settings including initial nitrogen pools were assigned based on field data collected in Roland et al. (2013) and Nicklen et al. (2019). We also follow conventional forest gap model practice by starting all of our simulations at bare ground.

### 2.3 Sensitivity analysis

To fully emulate UVAFME using a Gaussian process surrogate, we considered all data inputs and data outputs to create a statistical representation of the mathematical model. UVAFME has 23 species parameters, 24 meteorological drivers per year run, and 10 site‐specific conditions that are defined at the onset of the modeling process as inputs. Individual growth is incremented annually, and there are also between 10 and 100 belowground pools that are updated. For a 100‐year computer model simulation, there are 2456 inputs (= (23 × 2) + (24 × 100) + 10) and between 1000 and 1,000,000 outputs depending on the number of individuals simulated. It is not pragmatic to vary each input individually to adequately represent all dimensions of the model. We must reduce the dimensions of the problem to gain meaningful ecological inference. For our purposes, we are interested in learning about parameters driving competition between spruce species, so we omit meteorological drivers and model input settings hereafter. We performed a sensitivity analysis over the species‐level parameters to determine the parameters that were most important to constrain. Sensitivity analysis is a common method for reducing model dimensions by choosing parameters that are most likely to affect the output of interest (LeBauer et al., 2013).

We performed a sensitivity analysis on parameter sets for *P*. *mariana* and *P*. *glauca* in UVAFME. To do this, we ran eight simulations for each of the 23 parameters for each of the two species, varying one parameter at a time across an informative uniform prior centered on default values with width set based on expert knowledge of the range of realistic values from Foster et al. (2019). This resulted in 85,744 forest simulations (= 2 × 23 × 8 × 233) across 233 sites in Denali National Park. Starting at bare ground, we ran each simulation for 500 years to span the longest tree‐ring record (346 years). Across each species set of simulations (*n* = 42,872), we calculated the first principal component score for each basal area time series using the “princomp” function in the R computing environment (R Core Team, 2019; Venables & Ripley, 2013). See Appendix A8.2 for pseudocode. We then determined the parameter sets that were most sensitive across sites by calculating which parameter had the largest variance across first principal component scores by parameter at each site. We chose the first principal component score as our sensitivity diagnostic because it is a simplified representation of the shape of the trajectory of basal area over time. The parameter that most changed the first principal component score variance and therefore the simulation trajectories was identified as the most sensitive for a given site–species combination.

### 2.4 Model calibration with a Gaussian process surrogate

We estimated species‐specific parameters for the most sensitive parameters at each coexistence site using tree‐ring data to calibrate UVAFME and accurately simulate competitive dynamics between *P*. *glauca* and *P*. *mariana* over a centennial time frame. We did this with three steps listed here and described below: (1) Input grid—we simulated forest trajectories across a grid of the most important parameters determined by the sensitivity analysis using UVAFME at each site; (2) optimal coring year—we determined the best set of trajectories by calculating historical basal area trajectories for an alive tree subset each year of the simulation and using model selection to determine the optimal coring year in the simulations; (3) parameter estimation—we fit a surrogate model to the alive tree subset at the optimal coring year and estimated the best parameter combination given the tree‐ring observations.

#### 2.4.1 Input grid

We determined the species parameter input grid for the simulations at each site using the most important site parameters from the sensitivity analysis. We varied parameters similar to the sensitivity analysis across a uniform prior centered on the species parameterizations with prior spread determined from expert knowledge from Foster et al. (2019). We selected 36 parameter combinations per site using a Latin‐hypercube sampling design implemented with the “lhs” R package (Carnell & Carnell, 2016; Lin & Tang, 2015). This resulted in 36 forest basal area simulations per coexistence site. We chose 36 parameter combinations after initial attempts with fewer simulations (e.g., 5 × 5 = 25) where our parameter estimation process resulted in poor basal area predictions. More simulations could be added in the future, but we did not add more simulations because we met our objectives with 36 simulations at each coexistence site for this example of model calibration.

#### 2.4.2 Optimal coring year procedure

**FIGURE 1:**
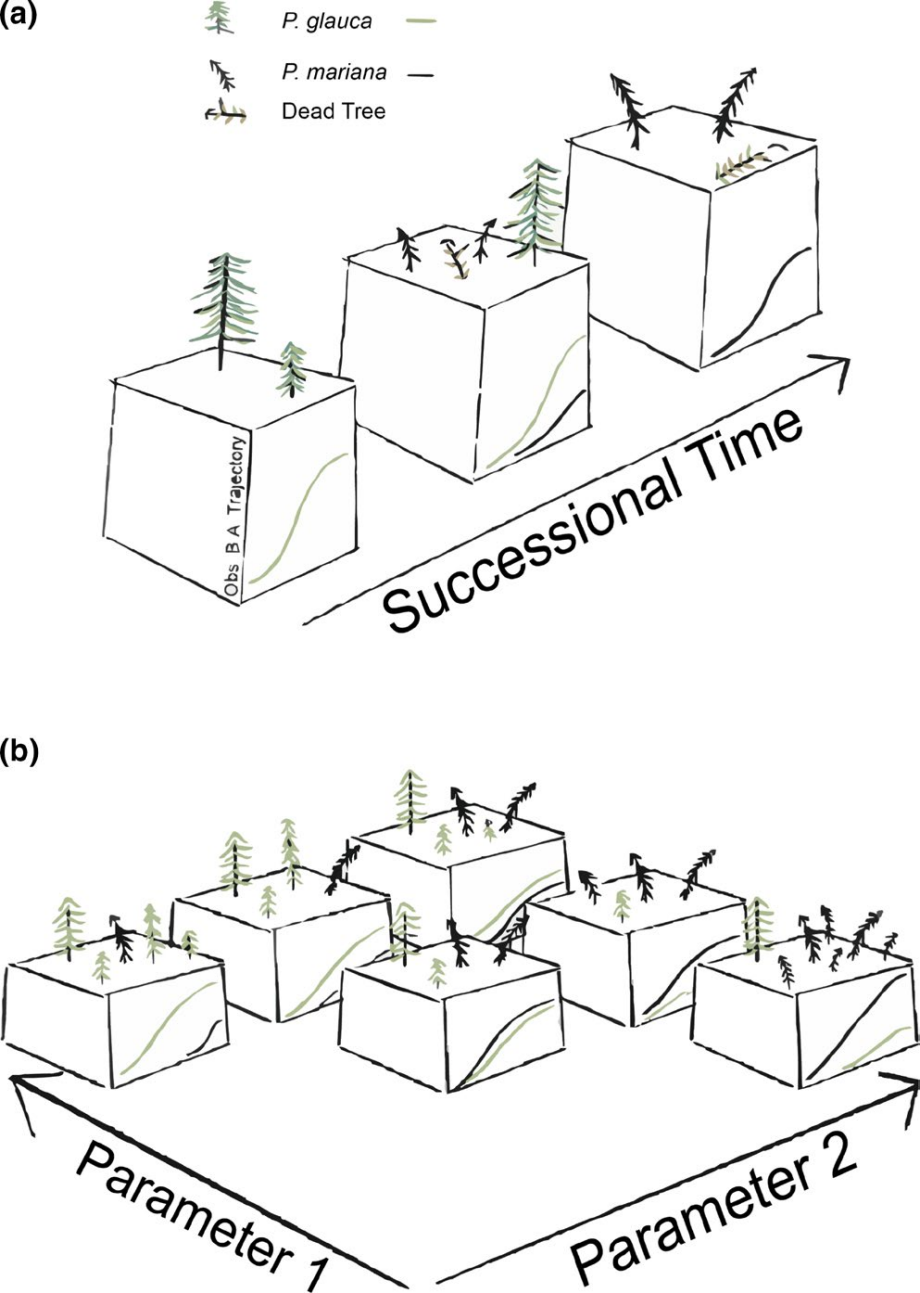
(a) Cartoon of one simulation of a forest stand at three time decadal steps t=1,2, or 3. In this example, *Picea glauca* is dominant at t=1, both species are present at t=2, and *Picea mariana* is dominant at t=3. Observed basal area trajectories of the alive tree subset for each species (black for *P. mariana* and green for *P. glauca*) are shown on the side of each cube and illustrate how the basal area trajectory observations may change depending on when the trees were cored during successional time. (b) Consistently labeled with cartoon (a). Each cube shows the alive tree subset basal area reconstructions for a given simulated forest stand. Forest stands vary with parameter sets used in the computer experiment

As discussed previously (Section 2.1), tree‐ring observation uncertainty includes uncertainty from the fading record where earlier tree rings are an inaccurate representation of forest stand basal area. Tree rings also provide the best data available for understanding long‐term competitive interactions between individuals. Dendroecologists have developed sophisticated statistical models for estimating the uncertainty associated with basal area reconstructions from tree‐ring records (Alexander et al., 2018; Dye et al., 2016) with the aim of incorporating these records into forest simulation models. While these methods are statistically robust and necessary for some data assimilation methodologies (Raiho et al., 2020), we used an alternative approach where we sampled the data and the model using the same methodology. As discussed in Section 2.1, we summed across the basal area increment measurement assuming that all trees were measured within a plot. In accordance with this assumption, we also thinned the model output to match the data‐generating process where, at each time step, we calculated the basal area trajectory only for trees that would have been alive and large (>10 cm) when cored (i.e., the “alive tree subset”; Figure 1a). This subset varied with coring time because we assumed that the observation year, while known in calendar date, was unknown in successional time. This is similar to system proxy models used in paleoclimatology (Evans et al., 2013) and remote sensing (Jacquemoud et al., 2000) that are created to simulate an observable proxy from mathematical model output. We highlight that UVAFME has a mechanistic basis for constructing tree‐ring observations from the model output where the base mechanism is annual individual diameter increment given limiting growth factors determined from the environment (see Section 2.2).

We focused on calibrating the forest simulation model using tree‐ring data. To do this, we determined the optimal coring time within all model simulations according to the alive tree subsets. We consider optimal coring time as the simulation year at which the alive tree subset historical BA trajectories best match the tree‐ring BA reconstructions. We fit generalized additive models (GAMs; Hastie, 2017) to the tree‐ring‐derived basal area data by species using UVAFME species basal area model output as a predictor and additional temporal smoothing to account for residual dependence in the data. Our predictor variable was comprised of the alive tree subset for each species resulting from the UVAFME simulation output based on the simulated coring year across the 500 simulation years. We scored these models based on predictive ability (using AIC; Akaike, 1973) to identify the optimal simulated coring year for each site. We relied on these years when making statistical inference at the next stages of our modeling procedure. This process allowed us to find the historical trajectories that most closely matched our data so that we could focus statistical power on parameter inference using the surrogate model. In a fully Bayesian setting (Berliner, 1996), the initial conditions would be determined based on model‐data feedback during the model‐fitting process. As discussed earlier, this is not an option in our example because of complexity in the simulation models. Another option would be to include optimal coring time in the model calibration process, but because we modularized our process as described in the next section, we illustrate a pragmatic method for determining initial conditions and leave estimating initial condition start time as a future direction.

#### 2.4.3 Parameter estimation

Our aim is to provide an ecological example of a commonly used surrogate modeling (Bayarri et al., 2009; Higdon et al., 2008; Tuo & Jeff Wu, 2016; Tuo & Wu, 2015) known as the “Kennedy and O'Hagan” framework (Kennedy & O'Hagan, 2001; Liu et al., 2009), building on the surrogate modeling strategy Gramacy (2020). The basic premise uses a Gaussian process model (also known as “kriging”), which focuses the majority of statistical learning on the covariance matrix such that

(1) Y∼MVN(0,Σ)

(2) $$\Sigma =\exp\left\{‐\sum_{k=1}^{p}\frac{{\left(x_{k}‐x_{k}^{\prime }\right)}^{2}}{\theta }\right\}+\nu I_{x=x^{\prime }}$$

where the mean of the response variable (*Y*) is assumed to be 0, and the covariance is a function of the distances between the inputs with range (*θ*) controlling smoothness and nugget (ν) controlling the size of the discontinuity. While our modeling experiments are deterministic, see Lee et al. (2011) for nugget recommendations in surrogate modeling experiments. We use a Gaussian process in two different parts of the model calibration process described below.

At each of the 10 coexistence sites, we estimated two UVAFME model parameters, one for each species. This algorithm assumes the forest model simulations ($y^{M}\left(\cdot ,\cdot \right)$) are deterministic and underlie the observations in the field data ($Y^{F}$) with an estimated bias (b(·)) and process variance (ϵ) such that,

(3) $$Y^{F}=y^{M}\left(\cdot ,u^{*}\right)+b\left(\cdot \right)+\epsilon ,$$

where $\epsilon ∼N\left(0,\sigma_{\epsilon }^{2}\right)$, and $u^{*}$ is an input parameter value. We repeated the following steps over a full grid (i.e., 20 × 20) of possible parameter values at each coexistence site. We chose a 20 × 20 grid to increase the simulation parameter grid resolution (*N* = 36 to *N* = 400) and demonstrated the ability of this approach to estimate parameters with only a small subset of simulations (i.e., 9% of full grid). First, we built the surrogate of the forest simulation model (${\hat{y}}^{M}$) by fitting a Gaussian process to the input ($X_{n_{M}},u$) and output ($Y_{n_{M}}$) of the model experiments ($n_{M}=1,\ldots ,N$, N=36). Specifically, we fit a Gaussian process model with heteroskedastic noise to represent the increasing variability over time in the forest model simulations using the Matern covariance structure (Binois et al., 2018). Second, we made a prediction (${\hat{Y}}_{n_{F}}^{M|u}$) from the surrogate given a set of parameters ($u^{*}$). Third, we calculated the residuals between these predictions and the tree‐ring data (i.e., ${\hat{Y}}_{n_{F}}^{M|u}‐Y^{F}$). Fourth, we fit another Gaussian process model to these residuals to estimate bias ($\hat{b}$) to calculate the negative log likelihood of the parameters ($u^{*}$) given the bias. In this step, we were simply optimizing to minimize bias. Finally, across the 20 × 20 parameter grid, we determined which parameter set has the lowest negative log likelihood. From this procedure, we obtained a 400 pixel parameter likelihood surface, the most likely parameter combination ($\hat{u}$) according to the data, and the data‐estimated model bias correction ($\hat{b}$). With the estimated bias correction, we also obtained a bias square error estimate from the residual prediction and were able to show a bias‐corrected basal area prediction by adding the prediction from the original surrogate to the bias. We repeated the steps across all coexistence sites to obtain optimal parameter sets for the most important species parameters determined from the sensitivity analysis. We specifically chose to use a maximum‐likelihood approach instead of a Bayesian approach to provide an ecological example of a thrifty model calibration using a surrogate model. In using maximum likelihood, we reduced our computational time 10‐fold, because these calculations take only a few minutes or seconds on a personal computer. We implemented the statistical surrogate model using the “laGP” package version 1.5.5 and the “hetGP” package version 1.1.3 in the R computing environment version 3.6.2 (Binois & Gramacy, 2019; Gramacy, 2016; R Core Team, 2019b). We also provide pseudocode in Appendix A8.2.

## 3 RESULTS

### 3.1 Sensitive parameters

**FIGURE 2:**
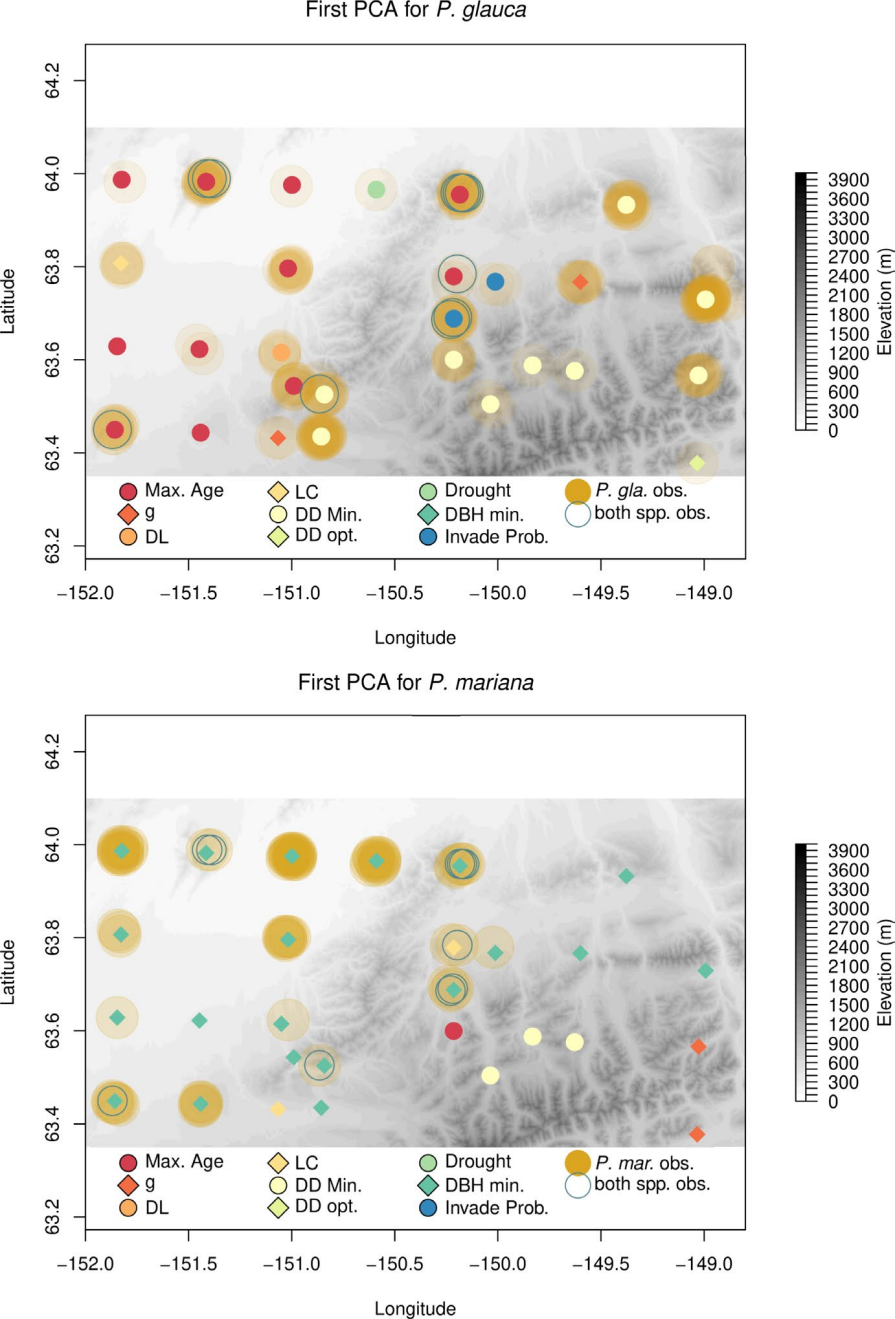
Elevation map of northern Denali National Park. Colored points show the most sensitive species parameter from the forest simulation model for each site cluster for *Picea glauca* (top) and *Picea mariana*. Tree‐ring observations are indicated behind the colored points in transparent yellow where increasing saturation indicates more observations of a particular species. Circles indicate the 10 coexistence site locations where both spruce species were observed when tree rings were collected. The most sensitive species parameters are as follows: life expectancy (Max. Age), growth parameter (*g*), scalar parameter for leaf area to diameter relationship (DL), leaf mass per unit area (LC), growing degree‐days minimum (DD Min), growing degree‐days optimum (DD opt.), drought tolerance (drought), diameter at breast height minimum growth increment (DBH Min), and probability of seed dispersal into the plot from outside the plot (Invade Prob.)

Parameters governing life expectancy (average maximum age (years), Max. Age) and species climate suitability (minimum growing degree‐days for growth; DD Min) dominated the sensitivity analysis of *P*. *glauca*. Max. Age is used in many of the allometric equations and is used to calculate an annual background mortality rate. If the growing degree‐days for a year (cumulative sum of degrees C above 5°C) are below DD Min for a species, the species DBH increment growth is 0 for the year. The parameter determining the minimum diameter increment before a tree is considered “stressed” and marked for potential stress‐induced mortality (DBH Min) dominated the sensitivity analysis for *P*. *mariana* (Figure 2). Trees have a chance (21%–33%) of dying if they have below‐minimum growth for at least 2 years. Across the 233 sites, the most sensitive parameters for *P*. *glauca* and *P*. *mariana* were life expectancy (Max. Age, 23% of sites) and minimum annual increment for diameter at breast height (DBH Min, 77% of sites). The second most sensitive parameters for *P*. *glauca* and *P*. *mariana* were growing degree‐days minimum required for growth (DD Min, 17% of sites) and leaf mass per unit area (LC, 10% of sites). Both sensitivity analyses showed spatial patterns from lowland (northwest) to higher elevation sites (southeast) where both species were sensitive to minimum growing degree‐days at higher elevations. The coexistence sites (Figure 2, open circles) showed somewhat different patterns for *P*. *glauca* with six sites having the maximum growth parameter (*g*) as the most sensitive parameter. The maximum growth parameter (*g*) determines the speed of initial DBH increment growth. Thus, faster‐growing species are typically parameterized to have a higher *g*. Over all 233 sites, *g* was the most sensitive parameter for only 14% of sites for *P*. *glauca*. Sensitive parameters for the coexistence sites for *P*. *mariana* showed similar patterns to the overall dataset with seven of the 10 sites having minimum annual increment for diameter at breast height (DBH Min) as the most sensitive parameter. Of the 6 coexistence sites where *g* is the most sensitive for *P*. *glauca*, 4 of them also had DBH Min as the most sensitive parameter for *P*. *mariana*.

### 3.2 Optimal coring times

**FIGURE 3:**
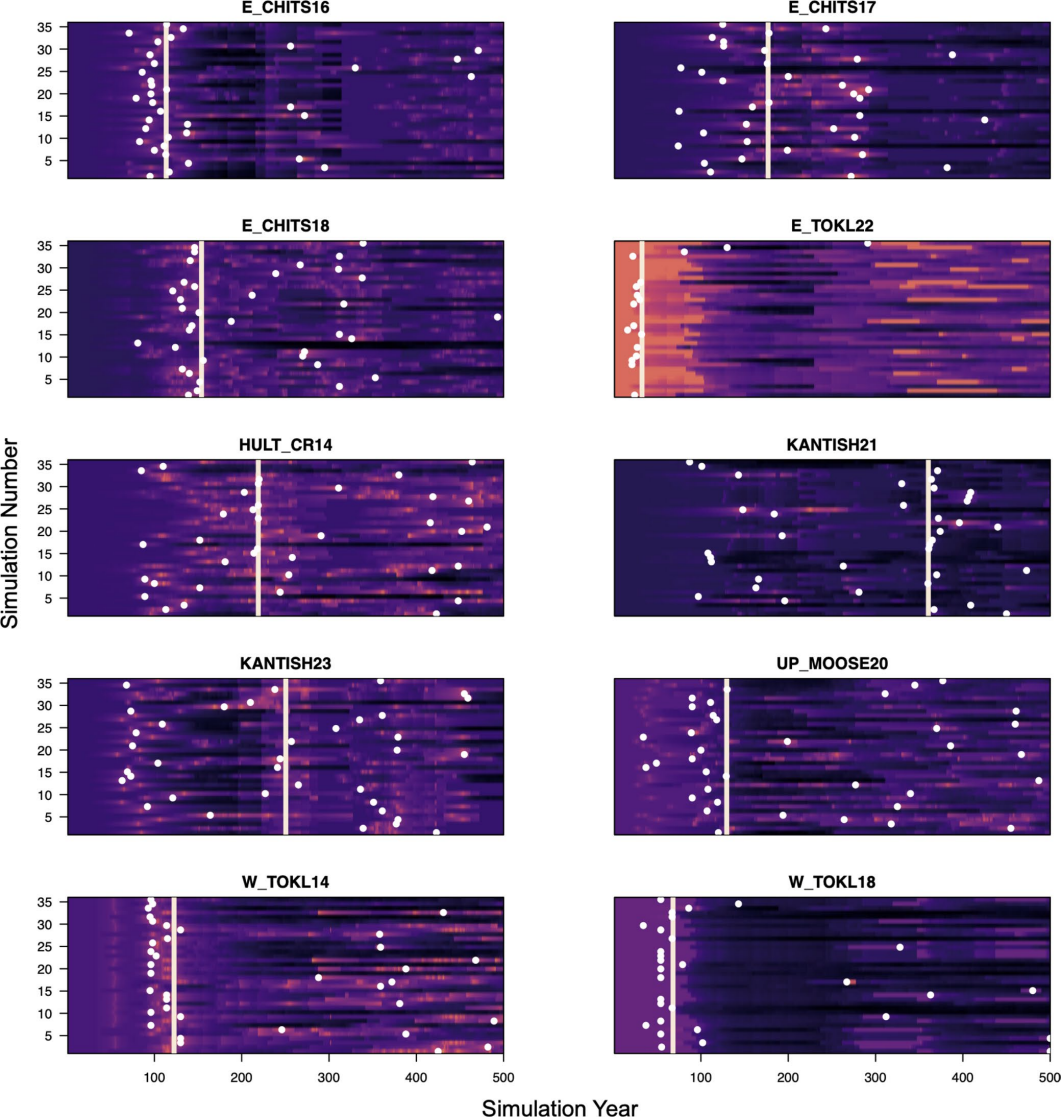
Optimal coring time results at the coexistence sites. White points represent the lowest AIC values for each simulation at a given site. Vertical white lines represent the median of the white points also called the optimal coring time. Plots are colored by AIC values with lighter colors representing lower AIC values. AIC color palette is relative to each site

**FIGURE 4:**
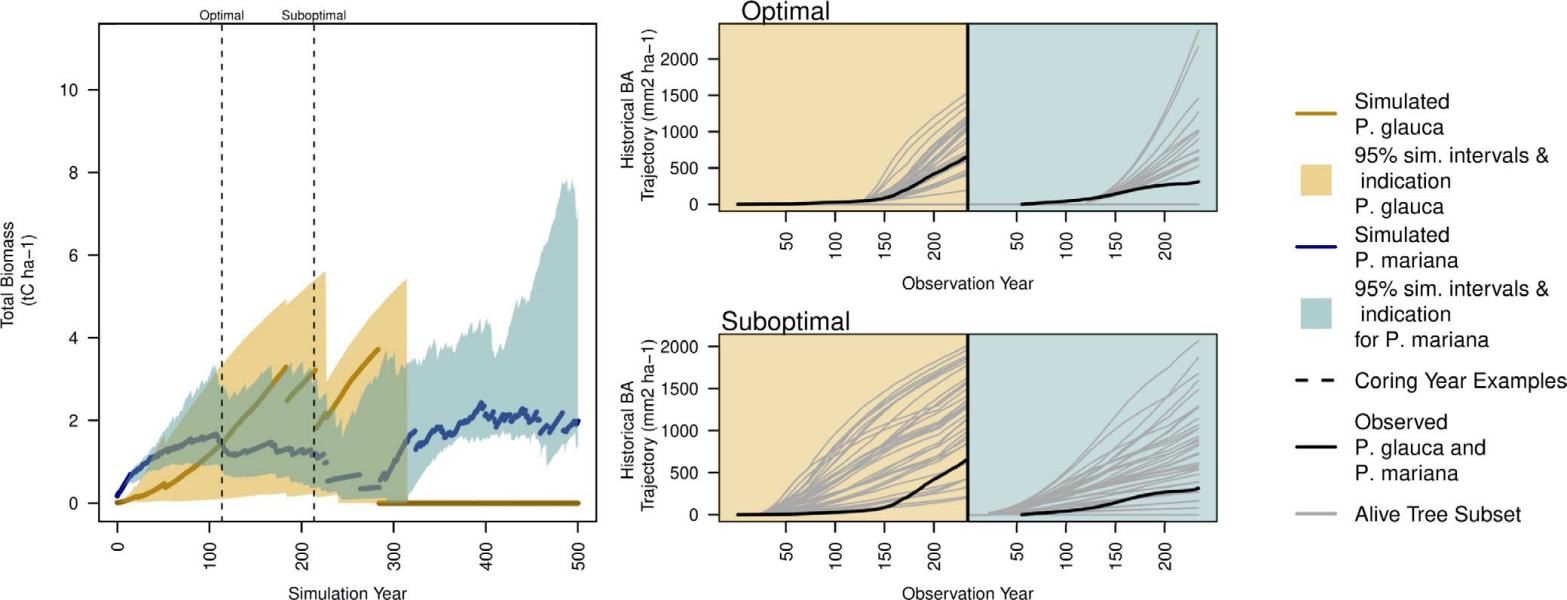
Left: Biomass trajectories over a 500‐year simulation for *Picea glauca* (yellow) and *Picea mariana* (blue). Line represents median across biomass trajectories, and shading represents 95% quantiles. Vertical lines represent optimal coring year chosen by the model selection procedure and example of suboptimal coring year. Right: Alive tree subsets (gray) overlaid with field‐collected tree‐ring data (black). The top plot represents the optimal alive tree subset collected from model output in year 113 of the model simulation, while the bottom plot represents a suboptimal example of the alive tree subsets collected from the model output in year 213

The majority of forest simulation site–year combinations at the coexistence sites showed *P*. *mariana* dominance over *P*. *glauca* (95.4%). This was caused by a combination of poor recruitment by *P*. *glauca*, nitrogen limitation (Figures A10 and A11: Appendix S1), and maximum age restrictions. However, *P*. *glauca* persisted in most model simulations between 0 and 300 years (Figure A12: Appendix S1). This pattern in the model output led to similar estimates of optimal coring years across sites (Figure 3 vertical lines, mean = 161.85 simulation year, SD = 96.3 simulation year) because, earlier in the simulations, both *P*. *mariana* and *P*. *glauca* were present in the model output. For example in Figure 4 left, at site E. CHITS16, *P*. *mariana* (blue) was dominant from year 0 to year ~120; then, *P*. *glauca* (yellow) became dominant from ~120 to ~280 on average across the 36 simulations for this site. During the optimal coring year analysis for E. CHITS16, we found that year 113 was the optimal coring year for model calibration. Figure 4 right top shows the alive tree subset for the optimal coring year contrasted with Figure 4 right bottom, which shows a suboptimal alive tree subset. Similar graphics for determining the optimal alive tree subset across sites is shown in Figure A12: Appendix S1.

**FIGURE 5:**
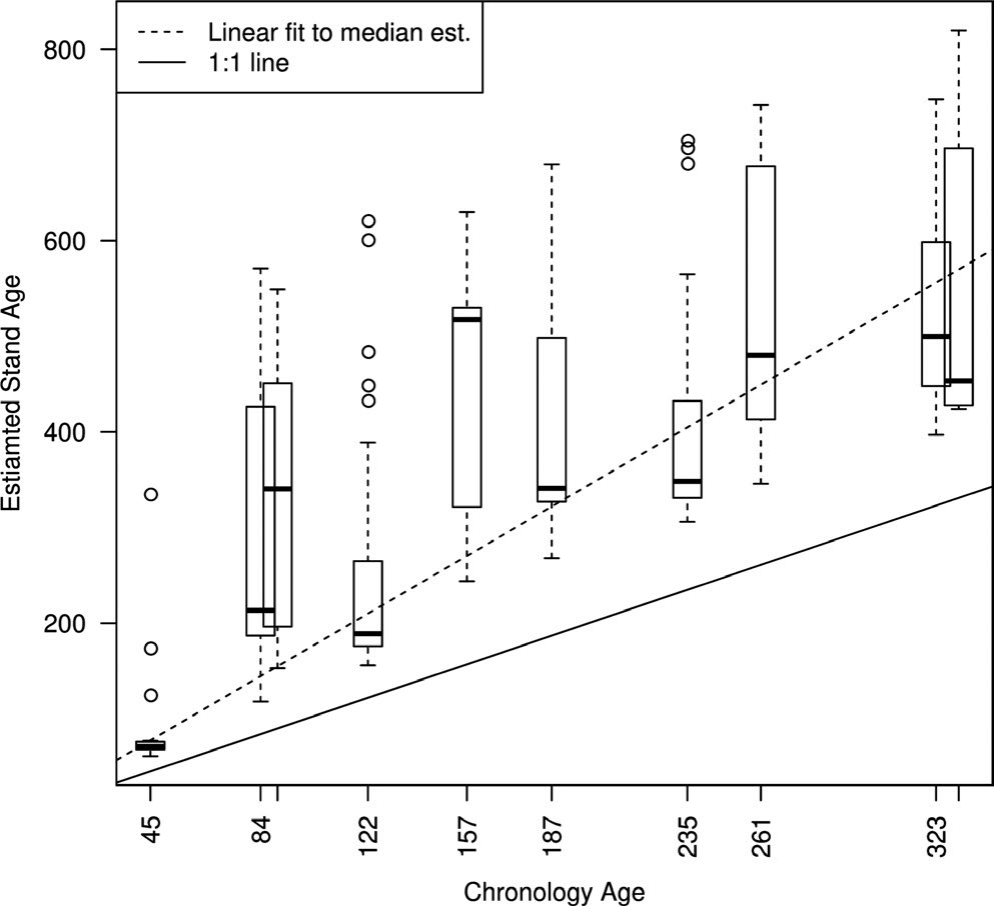
Chronology age determined by the number of tree rings collected at a site by estimated stand age determined by the optimal coring year selection process. Box plots represent uncertainty in estimated stand age between 36 simulations performed at each site. Solid black line indicates an assumption that chronology age and estimated stand age are synonymous, and dashed line represents the possible increasing bias in that assumption as chronology age increases

Using this process, we were able to estimate stand age. We found that stand age was related to chronology age, but as chronology age increased, the estimated stand age diverged from the chronology age (Figure 5, dashed line). This suggests that our approach may be able to account for bias in large tree sampling to improve estimates of stand age by reducing temporal bias (Gutsell & Johnson, 2002; Speer, 2010). However, this is a preliminary finding and more sites may be necessary to quantify the shape and magnitude of this bias.

### 3.3 Parameter estimates

**FIGURE 6:**
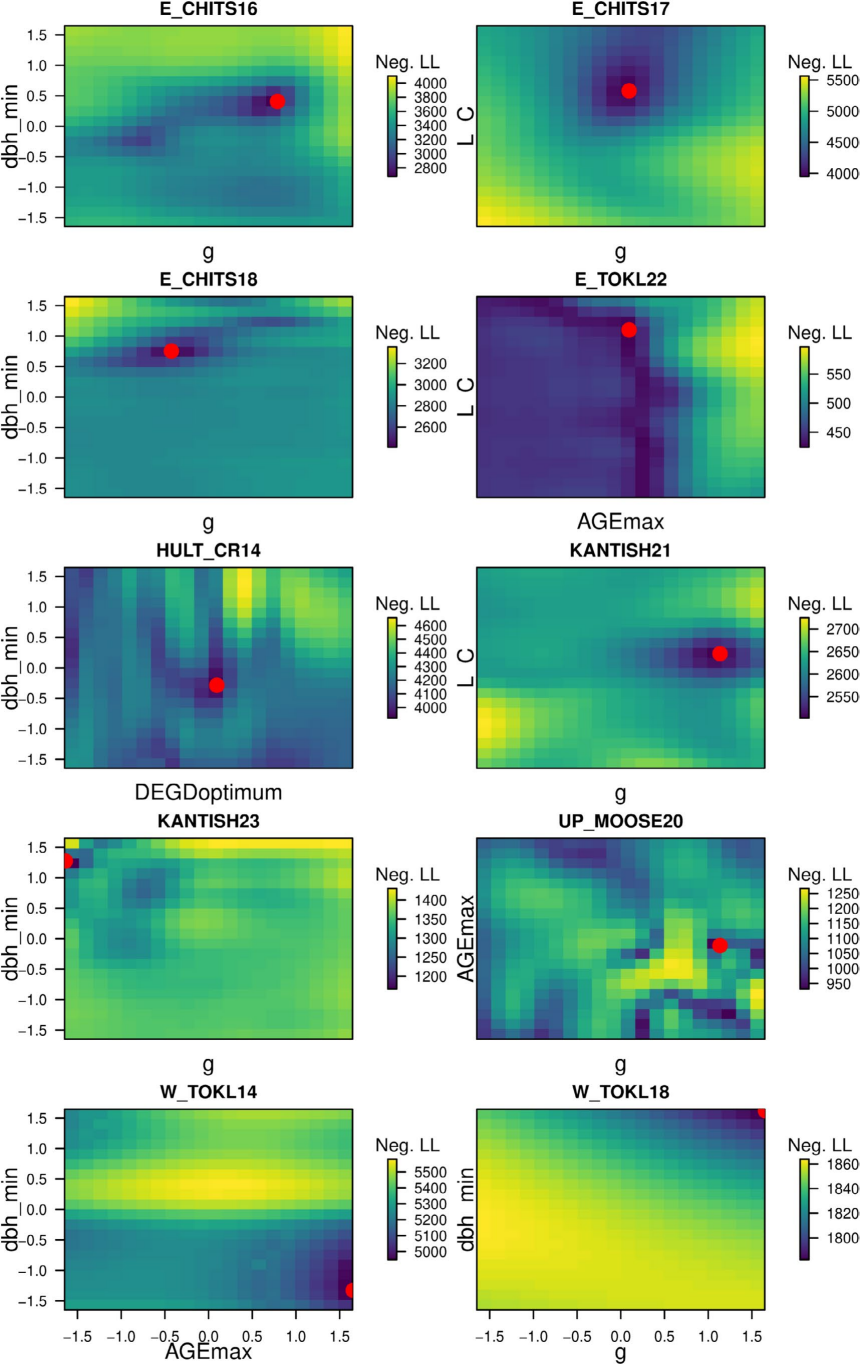
Negative log‐likelihood surfaces between the two most sensitive parameters for each site. Red points indicate lowest negative log‐likelihood value on each surface. Each figure is centered on the default parameter values

**FIGURE 7:**
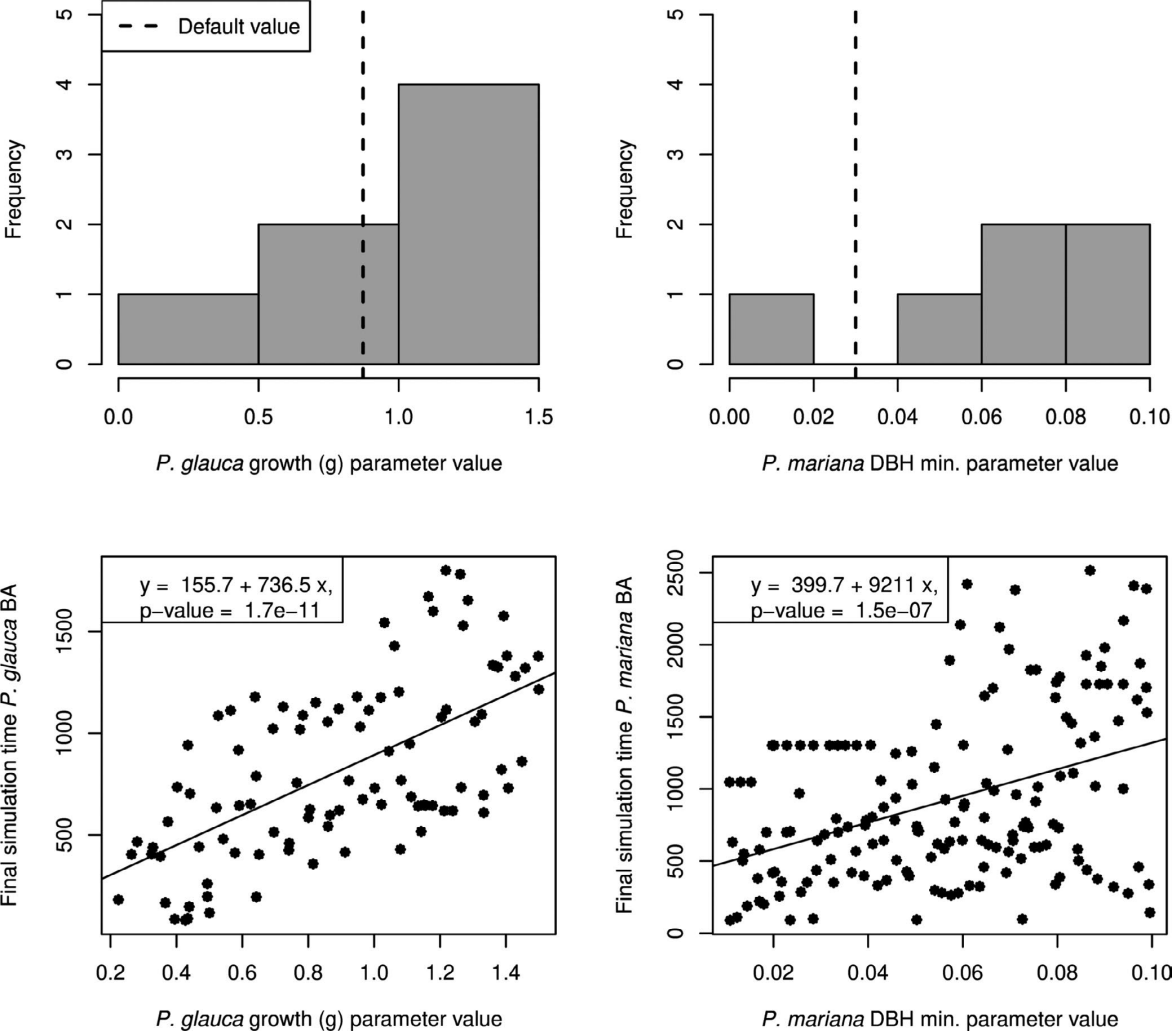
Top: Histograms of parameter estimates across coexistence sites from modularized Kennedy and O'Hagan algorithm (Liu et al., 2009). Default parameterization from Foster et al. (2019) shown as a vertical dashed line. Bottom: Relationship between chosen species parameter values and final basal area across simulations at coexistence sites where each point represents a single simulation. Diagonal lines represent fitted linear relationship with coefficient estimates shown in the top‐left boxes. Bottom left: The maximum growth parameter (*g*) affects the speed of initial DBH increment growth where higher values of *g* suggest faster‐growing trees. Bottom right: DBH Min determines the minimum diameter increment before a tree is considered “stressed” and flagged for potential stress‐induced mortality where higher values of DBH Min indicate lower stress tolerance

We estimated parameter likelihood surfaces (11× the resolution of the original simulations) at 10 sites for the two species most sensitive parameters using a GP surrogate for UVAFME (Figure 6). For the most sensitive parameters at the coexistence sites, neither resulted in parameter estimates near the default parameterization. We found that *P*. *glauca* growth parameter (*g*) was estimated to be high at the majority of the coexistence sites (Figure 7 upper left). We assessed the effect of *g* for *P*. *glauca* on the final basal model output area across the coexistence site simulations and found that increasing *g* values lead to increased *P*. *glauca* basal area in the simulations (Figure 7 bottom left). Higher estimates of *g* correspond to observations of tree‐ring data (Figure A9: Appendix S1, gray) that typically show greater *P*. *glauca* basal area than represented in the model simulations. Across the six coexistence sites where DBH Min was the most important parameter for *P*. *mariana*, none were estimated near the default value. Similar to *g* in *P*. *glauca*, DBH Min for *P*. *mariana* was typically estimated to be larger (Figure 7 upper right). However, the relationship between DBH Min and simulated *P*. *mariana* basal area is weaker than the relationship between *g* and *P*. *glauca* basal area (Figure 7 bottom right versus bottom left). The likelihood surfaces in parameter space (Figure 6) were multi‐modal and demonstrate that, while it is important to reduce dimensionality in the model‐fitting process, it may also be important to include additional parameters for these sites in the future.

**FIGURE 8:**
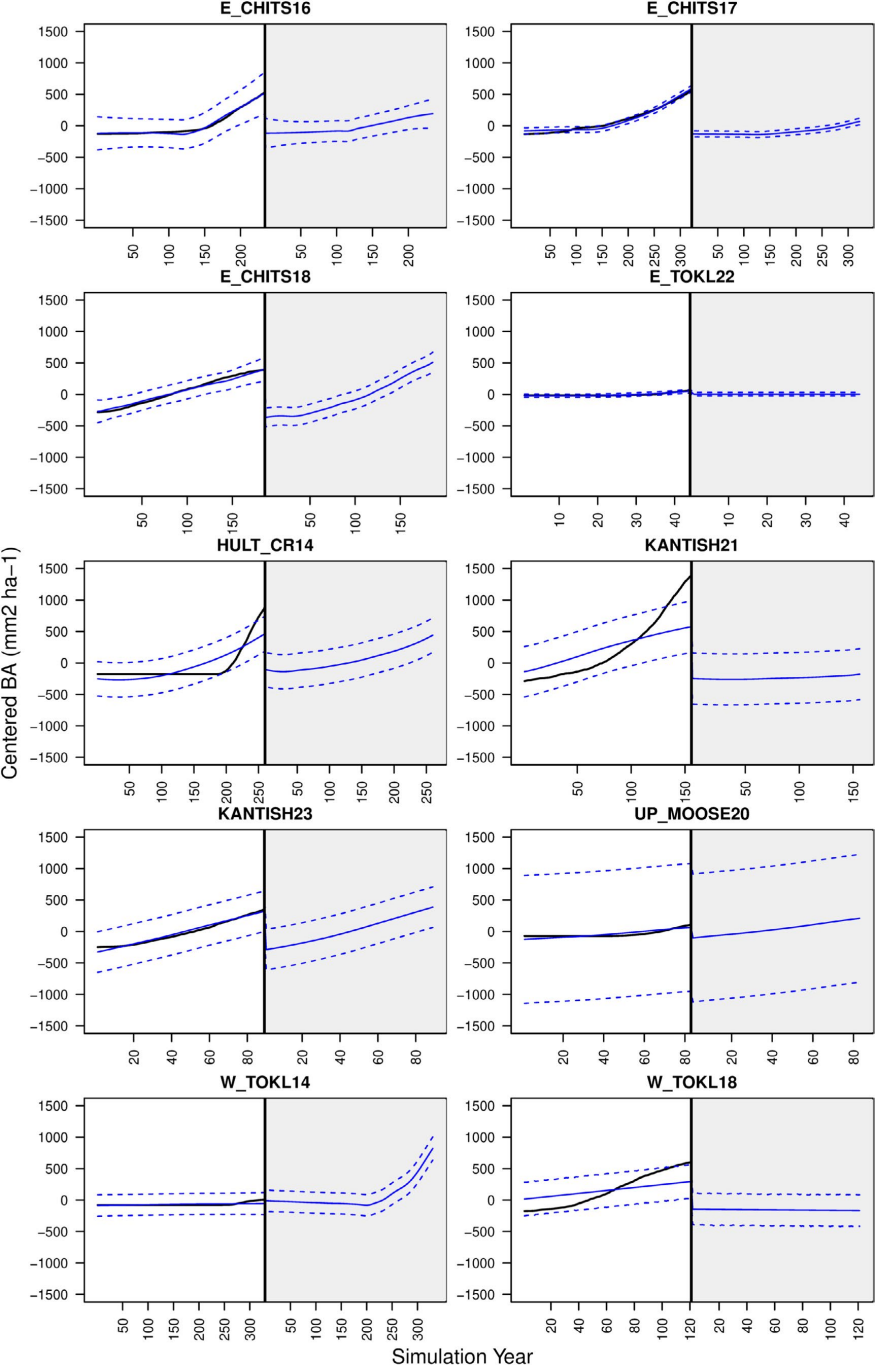
Adjusted model predictions (blue) via model GP surrogate with optimal parameters overlaid with data (black). Solid blue line is the median prediction, and dashed lines are 95% confidence intervals. Gray shading indicates *Picea mariana*, while no shading indicates *Picea glauca*

After determining the most likely model parameterization according to the data, we were able to adjust model predictions from the GP surrogate given information from the tree‐ring data (Figure 8). In some cases (Figure 8, top two rows), the optimal parameterization led to GP predictions that closely matched the data with low model prediction uncertainty, while in other cases (Figure 8, third and fifth rows), the predictions suggest that further model calibration may be necessary because there were both poor prediction using the optimal parameters and wide uncertainty estimates. In general, we found that model predictions were less similar to the data for *P*. *glauca* than for *P*. *mariana* (Figure 8, white versus gray) aligning with our results from the optimal coring years where coexistence of *P*. *glauca* and *P*. *mariana* was somewhat uncommon in our simulations because *P*. *glauca* was suppressed by poor recruitment and nutrient limitations.

## 4 DISCUSSION

DNPP faces climate changes that have been predicted to dramatically alter species composition and distribution (Chapin et al., 2004). *Picea glauca* is currently the most abundant tree species and may expand into *P*. *mariana* sites in the near future (Nicklen et al., 2021). Wirth et al. (2008) hypothesized that this expansion may cause a range contraction for *P*. *mariana* because *P*. *glauca* grows more quickly and may outcompete slower‐growing *P*. *mariana* during early post‐fire succession. However, *P*. *mariana* is also more tolerant of wet edaphic conditions and may itself expand into new sites (Roland et al., 2013). Currently, it is difficult to observe the competitive dynamics between *P*. *mariana* and *P*. *glauca* because they do not typically co‐occur (Nicklen et al., 2021). We used long‐term data to constrain the growth between the two species at sites where they do currently co‐occur within DNPP to better understand how they may affect each other in the future. Our model calibration results show that *P*. *glauca* may grow faster and *P*. *mariana* may become growth‐stressed sooner than current UVAFME default parameterizations (Figure 7) agreeing with assessments that *P*. *glauca* may have a competitive advantage over *P*. *mariana*. Many sites were estimated to be older than tree rings alone would predict (Figure 5). Similarly, across all 233 sites, the maximum age parameter (i.e., life expectancy) of *P*. *glauca* was the most sensitive model parameter. Emphasis on maximum age contradicts the assumption that early succession is the most vulnerable time for *P*. *mariana* in competition with *P*. *glauca* and suggests that understanding which environmental factors lead to long‐lived *P*. *mariana* may help predict future *P*. *mariana* distribution. Many experiments have been conducted that could inform these growth and longevity parameters under a variety of growing condition scenarios across sites (e.g., Johnstone et al., 2010).

Improving parameter representations may lead to better predictions from forest gap models and may also help ecologists pinpoint under‐studied mechanisms such as long‐term growth rate trade‐offs between species. For instance, *g* has not been significantly altered in UVAFME since its implementation in Botkin et al. (1972) and may have too strong of an influence over individual tree growth. Simulating coexistence between tree species is not specific to this model but is a difficult problem in global vegetation modeling in general because trade‐offs between species or plant functional types over time often result in one vegetation type dominating the simulation (Arora & Boer, 2006; Gravel et al., 2011; Turnbull et al., 2013). However, over the process of forest succession, species have opportunities to take advantage of different available resources (Falster et al., 2017). These successional trade‐offs are mechanistic and have been proposed as a path forward for improving global vegetation representation and predictions (Fisher & Koven, 2020), but the first step may be to understand which parameters are driving long‐term coexistence between species. While our example is not comprehensive, our approach provides a pragmatic path forward for improving multi‐species representations in vegetation simulation models using long‐term data.

Altering the model output allowed us to efficiently estimate model parameters while confronting uncertainties associated with the data‐generating process, in particular, the fading record (Babst et al., 2014; Nehrbass‐Ahles et al., 2014). In ecology, field observations are often proxies for the true quantity of interest. To accurately represent the true quantity of interest, ecologists have two options: alter the observation model to account for measurement error or alter the model output to match the observation. Both of these options involve modeling a translation between the field observation and the quantity of interest, which can be referred to generally as the “data model.” In our tree‐ring example, much effort has been put into constructing data models that translate tree‐ring widths into basal area or biomass “data products” to match forest simulation output (Alexander et al., 2018; Dye et al., 2016). Simultaneously, system proxy models in paleoclimatology or inverse transfer models in remote sensing have been developed to translate model output into observational proxies (Evans et al., 2013; Jacquemoud et al., 2000). We adopt this latter strategy and translate the individual‐level model output into species‐level basal area data to match the tree‐ring observations (Figures 1 and 4). This approach leverages the ecological mechanisms in UVAFME where each individual tree grows annual tree‐ring increments and allowed us to more easily determine which simulations to use for model calibration.

Our approach relies on simulations from a computer experiment spanning a range of output covering what was observed in the data. While our approach for estimating optimal coring time was pragmatic, sometimes (e.g., Figure 4 top right, *P*. *mariana*), the optimal alive tree subset did not fully overlap the historical trajectories from the data. The GP approach allowed us to interpolate these missing components of the simulations in most cases, but at some of the coexistence sites (e.g., UP. MOOSE 20), we may need to include more parameters when building the GP to fully calibrate UVAFME to simulate the dynamics between *P*. *glauca* and *P*. *mariana*. Iterative approaches have been suggested for vegetation model calibration (Fer et al., 2018; Hartig et al., 2011). A related procedure for performing optimal design of the computer experiment to identify the next set of simulations could be incorporated to improve the GP surrogate for a particular site (Williams et al., 2018). This approach has been useful in engineering (Ju et al., 2018) but remains to be tested in an ecological setting where model output and field data observations may be mismatched.

Like field data, most mechanistic model output does not represent the true process of interest. However, unlike field data, simulation models have the ability to represent many dimensions of the observable process, including missing data or emergent phenomena. This can make it difficult to determine which model subspace the data represent. In our example, the subspace of the modeled process that was difficult to determine was the coring sample date, which relies on knowing when the data were collected during the forest stand development process. Important influence of initialization and/or boundary conditions on prediction is a common property in ecological systems (Hastings, 2001). The general strategy in statistical models is to estimate the start or end time based on the data. Yet, embedding a mathematical model into a statistical model is not always pragmatic. A common method that simulation modelers use to confront this problem is to assume that the start time (t=1) is known or that the start time begins after an equilibrium state (Carvalhais et al., 2008; Elzein et al., 2020). For forest stand development, this assumption is met by initializing the model at bare ground and running the model for many years before analyzing the model output. Our approach shows that this assumption may be negatively biasing the stand ages in our example (Figure 5) and that the stand initialization time can be optimized using the data. A future direction of our approach is to include the optimal coring data within the model calibration procedure itself, but this approach would sacrifice computational time in the estimation process and may not yield different results. Many studies have found that forests are sensitive to initial conditions (Raiho et al., 2020; Temperli et al., 2013). Our approach provides a way to estimate stand initiation with tree‐ring data and forest simulation models together to leverage the long‐term observations while accounting for missing processes with a mechanistic model for forest stand development.

All simulations for this study were performed on a portable computer. This is atypical of an individual‐based model where computational times typically force the modeler to use distributed computing resources (Hooten et al., 2020). For the non‐specialist, this requirement may be prohibitive or discouraging, but it is possible to use our approach without performing the model simulations oneself. A surrogate GP may be fit to any inputs and outputs of a computer experiment and used to create an emulator for the computer model. Ecosystem model developers invest large amounts of time learning new coding languages, researching the history of simulation model development, and investing in understanding model intricacies. While model developers are extremely important to the continued improvement of the mechanisms within the simulation models, many research teams have pointed to the need for more multi‐model comparisons (Renwick et al., 2018; Thomson et al., 2006) and more sophisticated assimilation of field data (Fer et al., 2021). This type of work can be completed by a statistician or quantitative ecologist who may not have an interest in the development of a particular model code base. Beginning ecologists who have a specific interest in constraining many different types of simulation models with field data may find that exploring GP modeling with preexisting inputs and outputs available on public archiving sites is a more efficient entry point. This strategy may help create a larger workforce for fitting ecological models to field data by more efficiently introducing students to data assimilation without requiring them to perform the model simulations themselves.

Bringing computer simulation models together with field data has great potential for improving ecological inference and forecasting for a variety of applications (Christin et al., 2019; Dietze et al., 2018; Luo et al., 2011; Peters et al., 2014). In interior Alaska, rapidly improving understanding and quantitative predictions of forest distributions is necessary for ecological management under changing climate. Ecologists can increase the pace of model improvements by continuing to build on a growing suite of existing model calibration tools (Fer et al., 2018; Oberpriller et al., 2021; Pietzsch et al., 2020; Speich et al., 2021; Tao et al., 2020) and team members by trying new methods in different contexts. We provided the first example of a forest model calibration using tree‐ring data allowing us to demonstrate three pragmatic approaches to proceed with model calibration: connecting model outputs to data‐generating processes, determining data‐driven starting conditions from a suite of model simulations, and reducing a high‐dimensional model calibration problem *a priori* to model fitting for computational efficiency. These approaches are a subset of a growing toolbox for building robust connections between computer models and field data in ecology.

## AUTHOR CONTRIBUTIONS

**Ann M. Raiho:** Conceptualization (lead); formal analysis (lead); methodology (lead); visualization (lead); writing—original draft (lead); writing—review and editing (lead). **E. Fleur Nicklen:** Conceptualization (supporting); data curation (lead); formal analysis (supporting); visualization (supporting); writing—review and editing (equal). **Adrianna Foster:** Conceptualization (supporting); formal analysis (supporting); methodology (supporting); software (lead); writing—review and editing (equal). **Carl A. Roland:** Conceptualization (supporting); data curation (equal); funding acquisition (lead); project administration (lead); supervision (equal); writing—review and editing (equal). **Mevin B. Hooten:** Conceptualization (equal); formal analysis (supporting); funding acquisition (supporting); methodology (supporting); supervision (lead); writing—review and editing (equal).

## Supporting information

Appendix S1

## Data Availability

The data that support the findings of this study are available from the corresponding author upon reasonable request.
