# Supplementary material for: Bridging implementation gaps to connect large ecological datasets and complex models
Source: Ecol Evol. 2021 Dec 14;11(24):18271–87. doi: 10.1002/ece3.8420 (PMC8717344; doi:10.1002/ece3.8420)
Supplement: Supplementary file 1 — Appendix S1 [file ECE3-11-18271-s001.pdf]

A8 Appendix

A8.1 Tree ring data

Tree ring data were collected at 233 sites when trees larger than 5cm DBH were available. The sites used a circular sampling scheme with an inner circle (10m radius) and an outer circle (18m radius) at each site. The outer plot plot was divided into 4 quadrants and generally the largest tree, that was at least 5 cm in DBH, in each quadrant was cored. If no trees were available within the outer circle, trees were cored within a reasonable distance from the site and distances to site center were recorded. Of the 233 sites, only 10 contained one of each spruce species (Fig. A9).

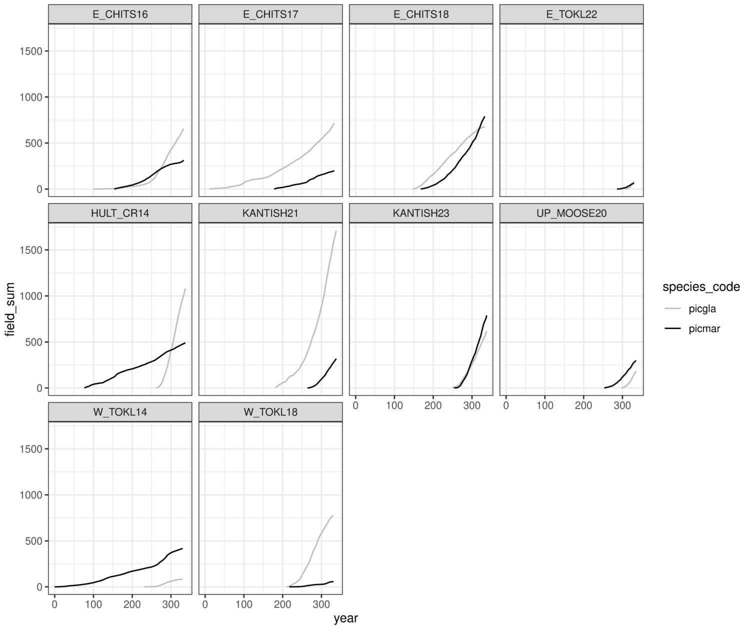

Figure A9: Basal area reconstructions from tree ring data across 10 sites where both *P. glauca* and *P. mariana* are present.

A8.2 Pseudo-code

Steps for PCA Sensistivity Analysis across each set (N = 8 x 23 = 184, with 233 sites x 2 species = 466 sets) of 500 year simulations:

1. Calculate PCA with princomp over time by simulation matrix
2. Extract PCA scores
3. Calculate the variance in the PCA scores for each parameter
4. Determine parameter that induced most variance in PCA scores
5. Repeat for each site and species combination

Steps for estimating parameters:

1. Computer Model Data
    (a) Select site and parameter runs of interest based on sensitivity analysis
    (b) Create input covariates (XU) with parameter and time columns to make GP univariate. We chose a univariate GP to maintain model fitting simplicity. However, we acknowledge that a next step may be to implement this procedure with a multivariate response variable.
    (c) Fit emulator to site stand level basal area from simulation experiments (yM).
        i. Use laGP::darg() and laGP::arg() to obtain starting values for computer model GP
        ii. Estimate nugget and lengthscale parameters with hetGP::mleHetGP(..., covtype = "Matern52"). While our model is deterministic, the individual based nature of the simulation model induces noise that is absorbed by the nugget. It is also recommended to include a nugget in model calibration exercises by Lee et al. (2011). Note: this step can take some computational time (i.e., not instantaneous).

iii. Assess fit with visualization of predictions in parameter and temporal spaces. Future applications may include some form of emulator validation at this step.

2. Calibration

   (a) Create parameter search grid over parameter and time space.

   (b) Loop over grid of parameter values to obtain most likely parameter estimate. The loop contains the following steps for each value in the grid:

      i. Use computer model GP to predict BA given parameter value with predict().

      ii. Compare those predictions to field data by fitting a separate GP to the difference between the field data and the simulation data with laGP::mleGPsep() and -laGP::llikGPsep().

   (c) Determine which parameter value has the lowest negative log likelihood that we call $\hat{u}$.

3. Bias We are able to solve for the bias (b) associated with the UVAFME model and shown in eqn. 3 by optimizing over the residuals at the best parameter set such that $-b(\cdot) = y^M(\cdot, u^*) - Y^F$ where u^* is the estimated optimal parameter set from the previous step.

   (a) Use calib() from above to fit a GP to the difference between the field data and the simulation data with $\hat{u}$ from the previous step.

   (b) From the GP fit to the difference, predict basal area $\hat{B}$.

   (c) Using $\hat{u}$ and the GP of the simulation data, predict basal area $\hat{M}$.

   (d) Calculate the bias corrected trajectory of basal area by adding the prediction from the computer model GP and the bias GP.

A8.3 Model output details

The model output in UVAFME is extensive and provides the modeler the ability to determine exactly why and how many trees are dying. Figs A10 and A11 shows *P. glauca* and *P. mariana* biomass loss respectively at the coexistence sites due to six factors that can cause tree mortality in UVAFME.

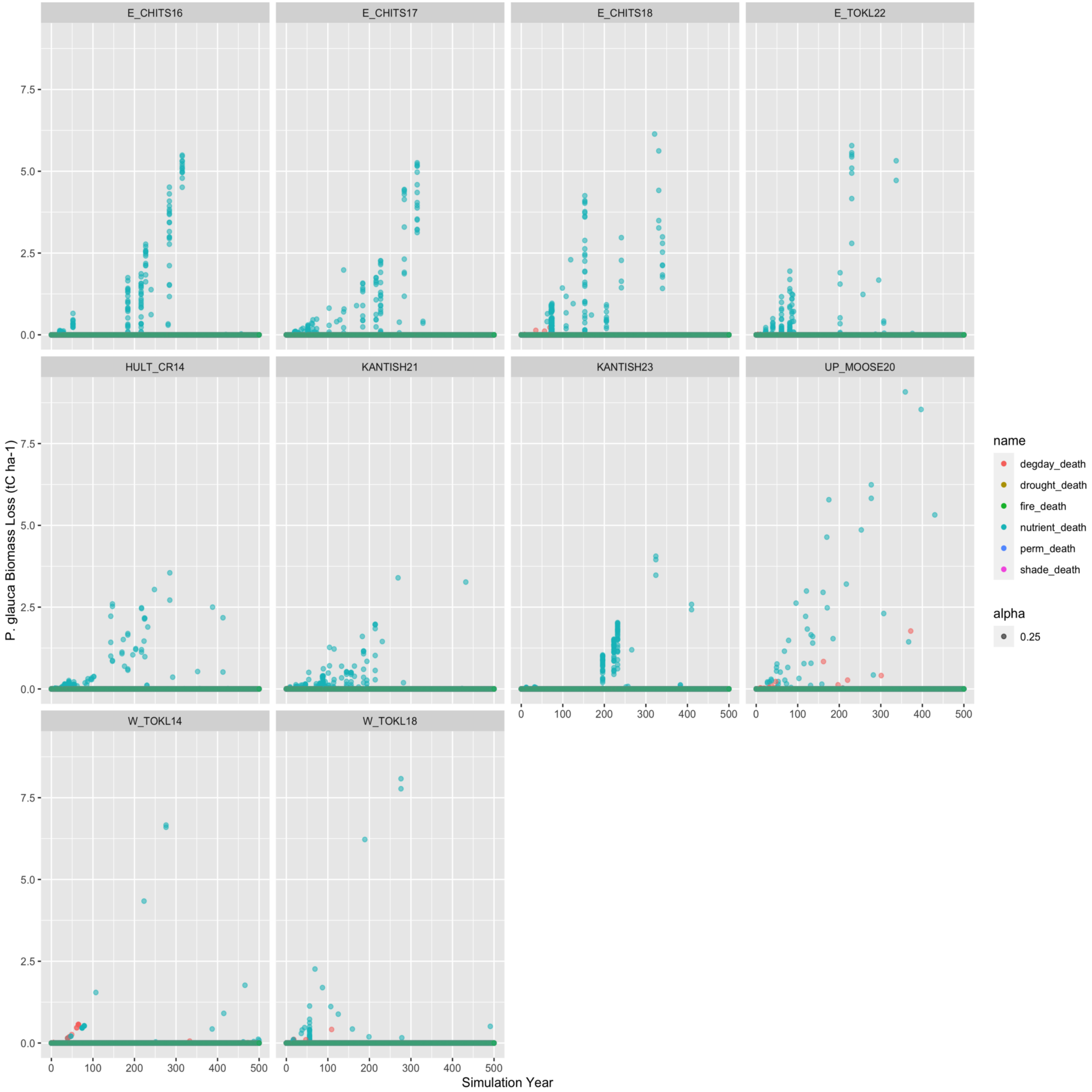

Figure A10: Scatterplots over time colored by the cause of biomass loss for *P. glauca* at the coexistence sites.

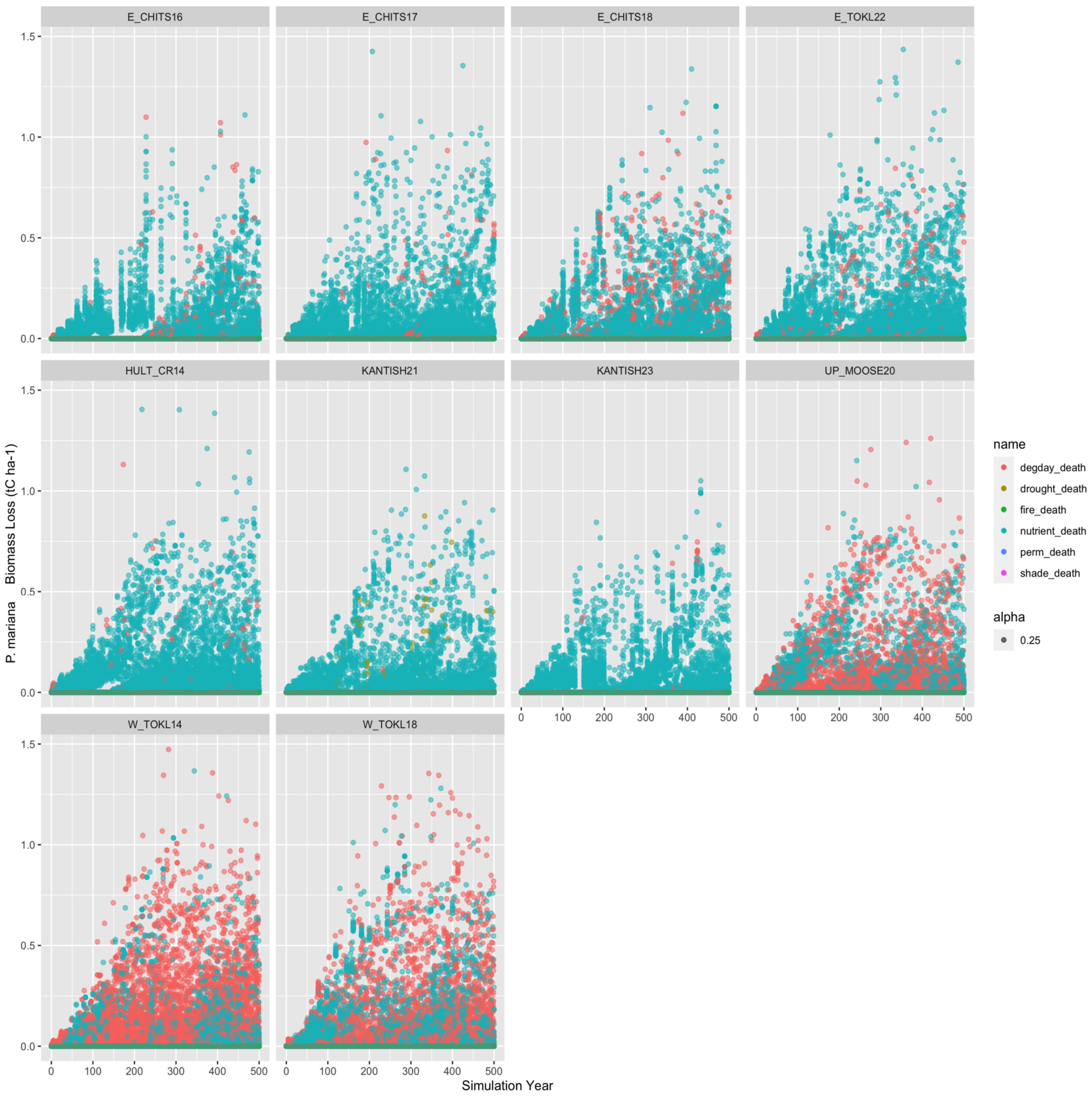

Figure A11: Scatterplots over time colored by the cause of biomass loss for *P. mariana* at the coexistence sites.

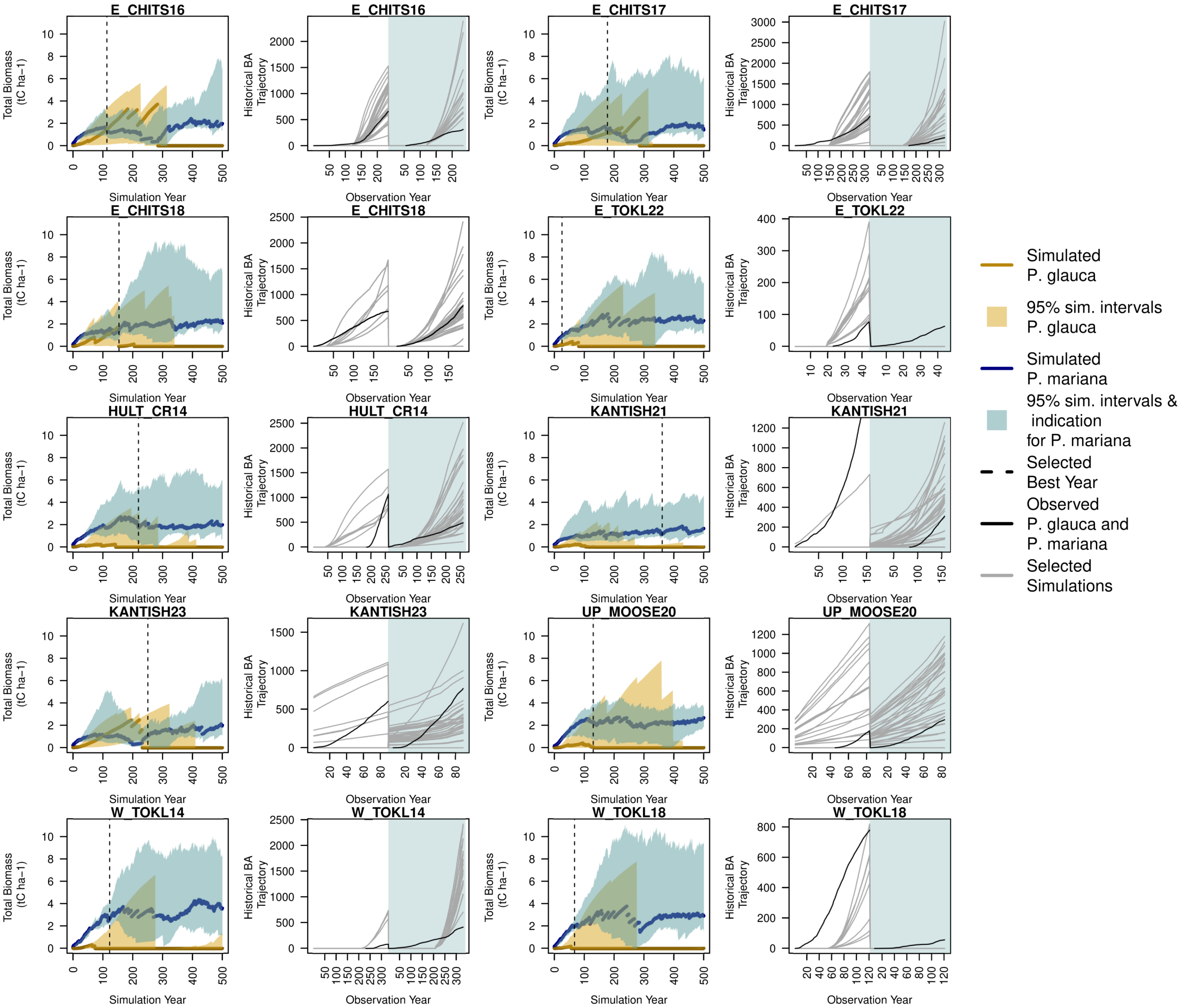

Figure A12: Model output and alive tree subsets for all coexisitence sites.
